# Anesthesia for Open Radical Retropubic Prostatectomy: A Comparison between Combined Spinal Epidural Anesthesia and Combined General Epidural Anesthesia

**DOI:** 10.1155/2019/4921620

**Published:** 2019-05-14

**Authors:** O. Kofler, S. Prueckner, E. Weninger, R. Tomasi, A. Karl, S. Niedermayer, A. Jovanovic, H. H. Müller, C. Stief, B. Zwissler, V. von Dossow

**Affiliations:** ^1^Department of Anesthesiology, Ludwig-Maximilians-University of Munich, Munich, Germany; ^2^Department of Anesthesiology, Ruprecht-Karls-University, Heidelberg, Germany; ^3^Institute of Emergency Medicine and Management in Medicine, Ludwig-Maximilians-University of Munich, Munich, Germany; ^4^Department of Urology, Ludwig-Maximilians-University of Munich, Munich, Germany; ^5^Institute of Medical Biometry and Epidemiology, Philipps-University of Marburg, Marburg, Germany; ^6^Department of Anesthesiology, Ruhr-University of Bochum, Bad Oeynhausen, Germany

## Abstract

**Background:**

Several anesthesiologic regimens can be used for open radical retropubic prostatectomy. The aim of this retrospective analysis was to compare the combined general epidural anesthesia and the combined spinal epidural anesthesia with regard to availability, efficacy, side effects, and perioperative time consumption in a high-volume center.

**Methods:**

A retrospective analysis was performed by querying the electronic medical records of 1207 consecutive patients from the database of our online documentation software. All patients underwent open radical retropubic prostatectomy from 01/2008 to 08/2011 and met the study criteria. Linear and multivariate regression analyses were performed to identify differences in parameters such as time consumption in the operating unit, hemodynamic parameters, volume replacement, and catecholamine therapy.

**Results:**

698 (57.8%) patients have been undergoing open radical retropubic prostatectomy under combined spinal epidural anesthesia and 509 (42.2%) patients by combined general epidural anesthesia. Operating unit (p <0.0001) and post-anesthesia care unit stay (p <0.0001) as well as total hospital stay (p <0.0001) were significantly shorter in the combined spinal epidural anesthesia group. In addition, this group had reduced intraoperative volume need (p <0.0001) as well as lower need of catecholamines (p <0.0001).

**Conclusions:**

This retrospective study suggests that the combined spinal epidural anesthesia seems to be a suitable and efficient anesthesia technique for patients undergoing open radical retropubic prostatectomy. This specific approach reduces time in the operation unit and length of hospital stay.

## 1. Introduction

Prostate cancer is currently ranked as the fifth position in cancer death and second in common cancer worldwide with 1.111.700 new cases each year. Prostate cancer is the most common cancer in males in developed countries (758.700 new cases each year) [1].

Radical prostatectomy is a primary treatment option for patients with clinically localized prostate cancer in all risk groups [2]. Open radical retropubic prostatectomy (RRP) (OPS-2014-5-604) is currently the most commonly performed surgical therapy for localized prostate cancer (ICD-10-GM-2014-C61) [3]. Extensive available data show a low morbidity for this type of surgery [4].

Various anesthetic techniques, i.e., general anesthesia only as well as combined general with epidural anesthesia and spinal anesthesia, can be successfully used to provide intraoperative anesthesia for RRP. General and neuraxial anesthesia (spinal or epidural) are generally equally efficacious intraoperatively. However, there is increasing evidence to prefer neuraxial procedures for urologic surgeries. For example, it has been shown that spinal and/or epidural anesthesia is feasible and is safely used for radical cystectomy for high risk patients with contraindications for general anesthesia [5]. Other studies indicate that the use of neuraxial anesthesia might be beneficial for reduction of intraoperative blood loss leading to reduced blood transfusion [6–8]. A large retrospective analysis suggests a beneficial effect of regional anesthesia on cancer progression after surgery for prostate cancer [9].

These findings have contributed to the discussion on the advanced value of neuraxial versus general anesthesia as well as impact on patient safety, and intraoperative processing times by different types of anesthesia [6, 7, 9–12].

Not only surgery, but also procedures and interdisciplinary aspects in the perioperative treatment process play a crucial role for successful treatment of patients [13]. Both the fast-track principle and the “Enhanced Recovery After Surgery” guidelines [14] aim to reduce perioperative stress and metabolic response by maintaining physiological body functions and rapid postoperative mobilization [15]. Consequently, postoperative morbidity remains low, leading to a shortened hospitalization period and a reduction in costs [13, 16].

Therefore, the major objective of this retrospective study was to compare two anesthetic regimes in a high-volume center for open radical retropubic prostatectomy with respect to anesthetic processing times. In addition, we performed an exploratory analysis of intraoperative hemodynamic side effects of the two regimes.

## 2. Methods

After approval by the Ethics Committee of the Ludwig-Maximilians-University of Munich (number: 313-11), we analyzed the medical records of all patients, who have been operated on with open radical retropubic prostatectomy in the Department of Urology at the Ludwig-Maximilian-University Munich between January 2008 and August 2011. This particular time frame was chosen, to ensure a study population of more than 1000 patients. Patients with prior prostate surgery or lymphadenectomy were excluded. The data analysis included all patients, treated via RRP with combined spinal epidural anesthesia (CSE) or combined general epidural anesthesia (CGE). Each patient was informed in detail about the advantages and disadvantages of both forms of anesthesia and the accompanying risk factors. The patients decided which anesthetic technique they preferred. Morbidity did not influence the choice of the anesthetic regime. In case of contraindication for regional anesthesia, the patients were excluded. All patients received midazolam 3.75-7.5 mg orally as premedication. Anesthesia was performed after inward transfer and patient preparation.

For CGE, the epidural catheter was punctured at the thoracic lumbar level (Th11/12 - L3/4, with preference to Th10/L1) and inserted 3-5 cm in the epidural space reaching the anesthetic level TH 10. After exclusion of an incidental spinal position of the catheter (test dosage 2 cc ropivacaine 1%), an initial dose of ropivacaine 1% (3-4 x 3cc) and sufentanil (10 *μ*g) were applied. General anesthesia was performed via peripheral line with “target controlled infusion” of propofol (plasma target 3*μ*g/cc) and remifentanil (brain target 3ng/cc). Prior to endotracheal intubation, neuromuscular relaxation was achieved by 0.15mg/kg cisatracurium.

Before the catheter was inserted in the epidural space (approximately 5 cm), CSE was induced using a needle-through-needle-technique on the lumbar level of L2/3 or L3/4 and initial dosage of isobaric ropivacaine 0.5% was injected intrathecally (3-5cc). Conscious sedation during surgery was obtained with intravenous midazolam (1-2 mg via bolus administration) or remifentanil (0,05-0,2 *μ*g/kg/min). The epidural catheter was loaded 60 minutes after insertion. In case of failed CSE, general anesthesia was induced.

Intraoperative monitoring included noninvasive blood pressure, heart frequency, respiratory rate, oxygen saturation, and temperature. In case of hypotension (MAP<65mmHg) continuous norepinephrine and in case of bradycardia (<50/min) 0.5 mg atropine were applied. Volume therapy was performed with isotonic crystalloid fluid about 3cc/kg/hour and additionally intraoperative blood loss substituted with colloid fluids.

Postoperatively all patients were monitored in the post-anesthesia care unit (PACU). Postanesthetic management of the patient included periodic assessment and monitoring of respiratory function, cardiovascular function, neuromuscular function, temperature, pain, mental status, nausea and vomiting, fluid assessment, urine output, drainage, and bleeding.

Postoperative pain management was achieved with continuous infusion (4-8 ml/h) of ropivacaine 0.2% in combination with sufentanil 1*μ*g/ml via epidural catheter. This was ensured normally for the next two days by using patient-controlled infusion pump (CADD ®, Smiths Medical, Minnesota). Therefore, an early mobilization was still guaranteed.

Perioperative time of anesthesia was documented and the individual time sections were compared. Observation time and software documentation (Narkodata, IMESO GmbH, Germany) ended with discharge from the PACU. Adequate vesicourethral anastomotic conditions in the cystogram and no pathological laboratory values were defined as patients discharge criteria.

The following perioperative parameters of 1464 patients were analyzed: demographic characteristics; perioperative anesthetic processing times, use, dosage and amount of atropine, norepinephrine, crystalloids and colloids and blood loss; perioperative time of anesthesia; anesthesia less surgery (=without surgical procedures); PACU stay and hospital stay. General anesthesia and the conversion of CSE to general anesthesia were applied as exclusion criteria.

### 2.1. Statistical Analysis

SPSS 20 (SPSS, Chicago, IL) was used for statistical analysis. In order to compare quantitative characteristics, mean values (standard deviations), median values (quartiles), and minimum and maximum values were calculated. If not otherwise stated, mean ± SD are displayed. All continuous data were tested for normality. The Wilcoxon-Mann-Whitney test was used for the comparison of process times, which are assumed to be not normally distributed. To compare data obtained from the two groups if deviations from normal distributions are not obvious, two-tailed Student's t-test for unpaired data was applied. To quantify the differences of the two groups concerning process times, the point estimates and 95% confidence intervals were provided. In case of nonparametric testing the method of Hodge-Lehman was used. P-value ≤ 0.05 was considered to be statistically significant at a nominal level, not adjusted for multiple testing. Multivariate regression models were constructed to adjust for possible confounding preoperative and intraoperative variables.

To adjust for multiple comparisons, statistical significance was considered at P < 0.05.

## 3. Results

**Figure 1:**
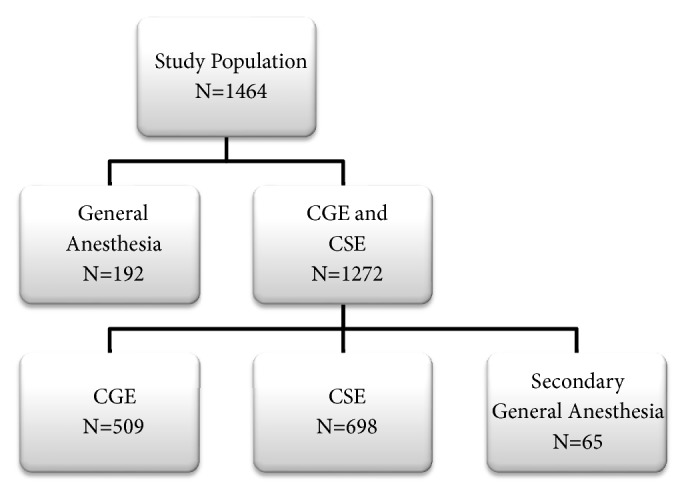
*The figure depicts the study population and subgroup distribution*: CGE: combined general epidural anesthesia; CSE: combined spinal epidural anesthesia.

In total, data of 1464 patients who received RRP without prior prostate surgery were screened retrospectively. We excluded 192 patients, because they did not meet the inclusion criteria, and 65 patients were excluded because of secondary conversion to general anesthesia. In total 1207 patients were included in our analysis with 698 patients receiving CSE and 509 patients CGE (Figure 1).

**Figure 2:**
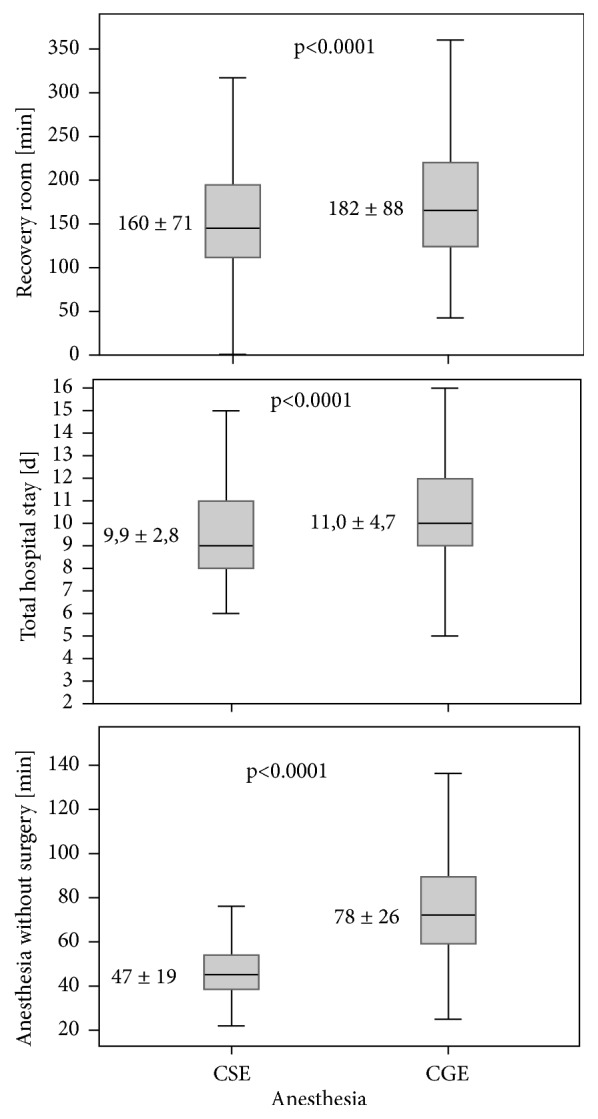
Total anesthesia without surgery, time in the post-anesthesia care unit and total hospital stay.

The time period between start of anesthesia (defined as first contact patient/anesthesiologist) and arrival in the PACU in the CSE group was 31 minutes shorter (47 versus 78 min). In our study, the CGE group attained the discharge criteria (Postanesthetic Aldrete recovery score) from the PACU 22 minutes after the CSE group (182 versus 160 min). Collectively, CSE resulted in significant shorter processing times compared to CGE (Table 1 + Figure 2). In addition, the hospital stay in the CSE group was significantly shorter (9,9 versus 11 days). (Table 1 + Figure 2).

Demographic data showed significant differences for age, body height, and ASA physical class between the groups (Table 1). The need of norepinephrine and the amount of atropine given were higher in the CGE group. The CSE group showed significantly reduced perioperative blood loss and less fluids were infused (crystalloids and colloids) (p <0.0001) (Table 1). Perioperative blood loss may have been affected by various surgeons. During hospital stay, no significant difference in mortality was noted between CGE and CSE group (0.14% versus 0.2%). The obtained significant results were analyzed in the second step by multivariate logistic regression. The following factors were considered in the multivariate logistic regression model: age, height, weight, ASA, blood loss, crystalloid, colloid, norepinephrine, atropine, and time of anesthesia. Crystalloids (p=0.006), norepinephrine (p<0.001), atropine (p<0.001), and time of anesthesia (p<0.001) were confirmed as significant confounding variables. A calculation of the CSE-group including the conversion group (65 patients) confirmed the same results in the multivariate logistic regression model.

## 4. Discussion

Open radical retropubic prostatectomy is an effective treatment for localized prostate cancer and for this indication the most common treatment performed worldwide [3]. To improve process quality and reduce perioperative costs, adequate anesthesiologic management is indispensable.

In our study, patients receiving CSE anesthesia had shorter anesthetic time, as well as in the PACU and hospital length of stay compared to the CGE group. Moreover, these patients needed less volume and catecholamine intraoperatively. Therefore, this retrospective study could show that CSE seems to be an efficient and suitable anesthesia technique with a significant reduction in perioperative anesthetic processing time and hospital stay.

Health systems are currently emphasizing the importance of cost reduction and transparency of surgical results. Enhanced-Recovery-After-Surgery (ERAS) pathways have been standardized using multimodal, interdisciplinary protocols that aim to improve surgical outcome by reducing variation in perioperative practice [17]. Initially described in the late 1990s [18], ERAS can accelerate postoperative convalescence, decrease costs, and maintain high quality [19]. The anesthesiologic setting plays an important role in the interdisciplinary process and in the implementation of the ERAS concept. Due to high incidence, prostatectomy patients have an enormous impact on perioperative costs. Therefore, the aim of this study was to analyze a few aspects of anesthesiologic intraoperative care components of ERAS in patient cohorts receiving CSE as an alternative to general anesthesia for RRP.

All patients in our cohort received epidural analgesia. In surgery, the benefits of epidural approach compared with systemic opioid analgesia are still controversial. Data show that patients with epidural anesthesia had significantly decreased risk of cardiac arrhythmia, deep vein thrombosis, respiratory depression, intubation risks, atelectasis, pneumonia, ileus, and postoperative nausea and vomiting [20]. Muscle relaxation for lower abdominal surgery is comparable to general anesthesia [21].

Reduced postoperative delirium in elderly patients is another benefit of regional anesthesia [22]. Patients with delirium are predominantly associated with increased mortality and high medical expenses. General anesthesia has been claimed as one of the most relevant risk factors for delirium due to physiological and psychological stress from pain, analgesia, and surgery [23–25]. In a parallel study with a similar patient collective, colleagues were unable to demonstrate a lower incidence of postoperative delirium in CSE (8.3 versus 7.7%) [26].

Among others, the time to emerge from anesthesia is affected by the choice of anesthetic agents and medications used in the perioperative period. Because of the absence of general anesthesia and the consistent presence of spontaneous breathing, our data demonstrate relevant shorter processing times in the CSE group compared to CGE. In addition, shorter postoperative monitoring times and early discharge from PACU are possible without general anesthesia. Concerning hospital stay, patients with CSE had an earlier discharge rate. The use of epidural anesthesia seems to have no impact on the hospital discharge.

In line with other published results comparing general versus spinal anesthesia, our cohort showed significantly reduced perioperative blood loss, reduced volume need, and lower need of catecholamines in the CSE group [12].

A common problem during RRP is the increased incidence of hemorrhage from the periprostatic venous structures. In comparison to general anesthesia alone, combining epidural with general anesthesia seems to reduce blood loss up to 35%, due to local hypotension and a pharmacological sympathectomy [11]. Venous blood pooling enhances these effects with decreased venous return and cardiac output, with the positive consequence of local hypotension in the surgical field [11]. However there are also published results showing no difference in anesthesia-dependent intraoperative blood loss [8].

Regional anesthesia reduces surgical stress response, which induces complex neurohumoral, endocrine, metabolic, and immunological changes. Nociceptive afferents and inflammatory mediators from the surgical area lead to increased energy consumption in a neuronal and systemic way resulting in catabolism and organic dysfunctions [27]. This biological cascade is responsible for perioperative morbidity and mortality. Local suppression of the stress response reduces the risk of perioperative complications [28].

Moreover, combining general anesthesia with neuraxial anesthesia for prostate surgery could positively influence cancer progression and overall survival. Data suppose a positive impact of anesthetic management (e.g., intrathecal opioids, local anesthetics) or mechanism (reduced stress response or reduced systemic opioids) which contribute to the apparent benefit [9].

A limitation of this study is the lack of randomization due to retrospective data collection. In addition, anesthesia with CSE is associated with a relevant conversion rate. In our population, 65 CSE procedures failed and had to be converted to general anesthesia. This corresponds to a ratio of 8.52% in the CSE cohort. In this group, the time period between start of anesthesia and arrival in the PACU without surgery was a mean of 76.6 min.

By reason of the study design, no conclusion could be drawn on the associated factors quoted as benefits to epidural anesthesia, i.e., cardiac arrhythmia, deep vein thrombosis, respiratory depression, intubation risks, atelectasis, pneumonia, ileus, and postoperative nausea and vomiting. Cancer characteristics were not questioned.

Our data collection ended with the discharge from the PACU. Postoperative pain monitoring on normal ward was not recorded in our study.

## 5. Conclusions

Based on a large number of patients included in this study, we were able to illustrate differences between CSE and CGE for RRP. Data demonstrate that CSE is an efficient and suitable anesthesia technique for RRP, resulting in lower perioperative demand for fluid and catecholamines as well as a faster processing time. This approach could be relevant in the perioperative treatment process. However we need to confirm our results in prospective, randomized studies.

## Figures and Tables

**Table 1:** Perioperative parameters.

	CGE	CSE	Difference (95% CI),
			method by Hodges Lehmann),
			P value
*Age (years)*	65.8 ± 6.9	64.7 ± 7.3	-1 (-2/ 0), 0.014
*ASA physical class*	2.1 ± 0.56	2.0 ± 0.57	0 (0/ 0), <0.001
*Body height (cm)*	175.4 ± 7.2	177.4 ± 7.7	2 (1/ 3), <0.001
*Body weight (kg)*	82.9 ± 11.8	84.1 ± 12.2	1 (0/ 2), 0.129
*Preoperative visit time (min)*	27.2 ± 6.6	26.9 ± 7.2	0 (0/ 0), 0.281
All results are shown as mean ± SD, p < 0.05 significant			

*Blood Loss (cc)*	569 (231-700)	355 (150-500)	-100 (-183/ -100), <0.001
*Crystalloid (cc)*	2775 (2000-3000)	2109 (1500-2500)	-500 (-500/ -500), <0.001
*Colloid (cc)*	1081 (500-1500)	830 (500-1000)	0 (-500 / 0), <0.001
All results are shown as mean ± SD, p < 0.05 significant			

*Norepinephrine max(mg/h)*	0.33 ± 0.53	0.06 ± 0.11	-0.20 (-0.20 / -0.20), <0.001
*Atropine(mg)*	0.17 ± 0.36	0.25 ± 0.4	0.00 (0.00 / 0.00), <0.001
All results are shown as median (range percentiles 25-75), p < 0.05 significant			

*Total Anesthesia Time (min)*	212 ± 72	117 ± 19	-79 (-84 / -75), <0.001
*Waiting Time (min)*	19 ± 12	16 ± 9	-3 (-4 / -2), <0.001
*Anesthesia less Surgery (min)*	78 ± 26	47 ± 19	-27 (-29 / -25), <0.001
*Post-Anesthesia Care Unit (min)*	182 ± 88	160 ± 71	-18 (-26 / -11), <0.001
*Total Hospital Stay (d)*	11.0 ± 4.7	9.9 ± 2.8	-1 (-1/ 0), <0.001
All results are expressed as mean ± SD, p < 0.05 significant			

## Data Availability

The Excel-File used to support the findings of this study is available from the corresponding author upon request.
